# An Increase in the Rigidity of the Environment Favors MLCT over the MC State in [Ru(bpy)_2_(Nicotine)_2_](Cl)_2_: A Case Study of Photolabile Ligands

**DOI:** 10.1021/acs.jpca.4c04914

**Published:** 2024-11-04

**Authors:** Mohini Semwal, Nikita Vashistha, Sven Rau, Benjamin Dietzek-Ivanšić

**Affiliations:** † Institute of Physical Chemistry,Friedrich Schiller University Jena, Helmholtzweg 4, Jena 07743, Germany; ‡ Research Department Functional Interfaces, Leibniz Institute of Photonic Technology, Albert-Einstein-Str. 9, Jena 07745, Germany; § Institute for Inorganic Chemistry I, Albert-Einstein-Allee 11, 89081 Ulm, Germany

## Abstract

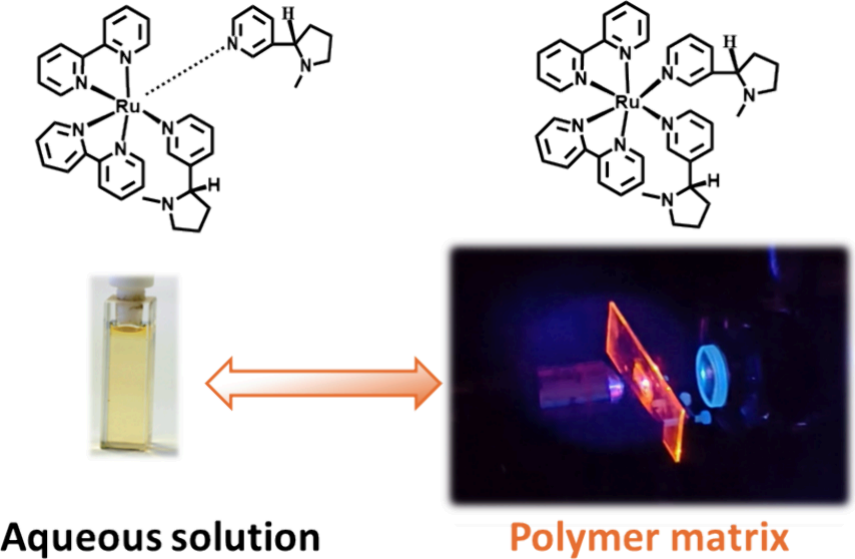

Ru(II)-complexes with photolabile ligands find a wide range of applications, e.g., in drug release and in the design of light-responsive interfaces. While light-driven ligand loss has been studied mechanistically in detail for complexes in solution, comparably few studies are present that investigate the process in a material context, i.e., in a rigid environment and in the absence of solvent. This paper adds to this underrepresented perspective by studying the excited-state dynamics of [Ru(bpy)_2_(nicotine)_2_] (Cl)_2_ (**Ru-nico**) as a model system in poly(methyl methacrylate) (PMMA) and polyacrylonitrile (PAN) matrices. Femtosecond transient absorption spectroscopy and time-resolved emission spectroscopy are employed to monitor the photodissociation of labile nicotine ligands in polymer environments. Photoexcitation within the metal-to-ligand charge transfer (MLCT) band leads to transient dissociation of the nicotine ligand when the complex is dissolved in water. However, optical excitation of the ^1^MLCT transition of the complexes embedded in polymer matrices does not result in photodissociation, likely due to the rigidity of the environment, which cannot solvate the undercoordinated complex after ligand dissociation and the dissociated ligand. These insights shed light on the role of the local environment when considering the photophysics of ligand loss from Ru(II)-polypyridyl complexes and, hence, their use in the light-activation of reactive molecular components in materials.

## Introduction

Ru(II) complexes with photolabile ligands have gained significant attention in recent years as model systems to understand light-driven ligand dissociation reactions.^1−3^ The light-induced ligand release from the complexes is key to a broad range of applications, e.g., in drug delivery, photodynamic therapy, sensing, solar energy conversion, and fabrication of molecular devices.^1−10^ To understand the molecular mechanisms underlying the specific function of the complexes, it is mandatory to amend structure–function relationships by spectroscopic studies revealing the light-induced dynamics in an environment, which is relevant to a specific application and, hence, specific to a certain function of the complexes.

When the functionality of Ru(II) polypyridine complexes hinges on their ability to undergo photochemical ligand loss, the design of the first coordination sphere is of utmost importance.^11−14^ In this context, commonly investigated ligands are polypyridine-based ligands, including pyridines, azapyridines, bipyridines, or terpyridine derivatives.^11,12,15−18^ Mechanistically, the photophysical behavior of such Ru(II) polypyridine complexes is governed by the interplay of metal-to-ligand charge transfer (MLCT) states with the MC states. These states are populated following metal-to-ligand excitation (MLCT) and intersystem crossing to the ^3^MLCT manifold by a thermally activated internal conversion.^19^ Once the MC states are populated, the complexes undergo either rapid nonradiative decay or, particularly when carrying monodentate ligands or strained bidentate ligands, ligand labilization and ligand-loss photochemistry.^13,20−24^ The use of strained ligands lowers the ligand field splitting energy of the complex, thereby stabilizing the nonemissive ^3^MC state over the ^3^MLCT state and in turn enhances the probability of ligand loss. Nonetheless, literature also reports ligand loss from Ru(II)-polypyridyl complexes directly upon MLCT excitation without previous population of ^3^MC states. In particular, Turro and colleagues as well as Bonnet and colleagues have studied the light-driven dissociation of nitriles and Hmte (2-(methylthio) ethanol) from ^3^MLCT states of Ru(II)-polypyridyl complexes in solution.^11,25−27^

As photodriven ligand loss from Ru(II)-polypyridyl complexes is associated with major intramolecular structural changes of the complexes, the rates and yields of the process do not only depend on the complex’s architecture, i.e., its first coordination sphere, but also on the environment, including the second coordination sphere into which the complex is placed. In particular, rigid media like frozen solvents, zeolites, porous glasses, and sol–gel matrices can stabilize the MLCT excited state by restricting the intramolecular reorganization of the complex upon photoexcitation and, hence, reduce nonradiative decay pathways and prolong the lifetime of the MLCT excited state.^24,28−37^ Recent detailed investigations point to the significant involvement of Jahn–Teller distortions involving significant Ru–N bond elongations involved in the population of the ^3^MC state.^19^ To understand the decisive role of the complexes’ environment, Meyer and co-workers elucidated the role of the environment in the ligand-loss photochemistry of Ru (II) complexes by embedding cis [Ru(bpy)_2_(py)_2_] (PF_6_)_2_ in a rigid poly(methyl methacrylate) (PMMA) film, giving rise to a switch-on of the emission of the complex.^33,36^ The authors rationalized their findings by an increased energy gap between the ^3^MLCT and ^3^MC states in PMMA due to intramolecular reorganization energy. This result points to the role of PMMA in inhibiting metal–ligand bond breaking leading to a (comparably) long-lived emissive state with an emission lifetime of 80 ns.^33^

Riehn and co-workers recently provided an intriguing study on the environmental impact on the photoinduced ligand-loss chemistry of Ru(II)-polypyridyl complexes by investigating the ultrafast dynamics of the Ru(II) nicotinamide complexes, i.e., [Ru(bpy)_2_(nicotinamide)_2_]^2+^ in the absence of any surrounding material, i.e. in the gas phase.^38^ The authors observed rapid (within 3 ps) dissociation of the nicotinamide ligand upon MLCT excitation, while the respective process took about 180 ps in solution. Riehn and co-workers rationalize this behavior considering a smaller energy gap between the ^3^MC and the ^3^MLCT in the gas phase, as the energy of the latter is raised in the absence of solvation.

The contribution presented here adds to the discussion of intermolecular control of light-driven ligand-loss chemistry of Ru(II) complexes. In particular, it will expand on the works by Riehn and Diller as well as Meyer in order to explore the light-driven reactivity of [Ru(bpy)_2_(nicotine)_2_] (Cl)_2_ (**Ru-nico**) in two different polymer matrices. Nicotine is a sterically more demanding and more electron-rich pyridine-type ligand compared to pyridine. Furthermore, it is also a biologically active alkaloid with a high biological activity, as it binds to nicotinic acetylcholine receptors in the brain. In addition to PMMA, we embed the compound into polyacrylonitrile (PAN) and probe the impact of both matrices on the excited-state dynamics by transient absorption spectroscopy and time-resolved emission spectroscopy. A comparative analysis with the solution phase revealed changes in the excited-state photophysics and chemistry of the **Ru-nico** complex. This became particularly apparent through the pronounced emissive behavior of **Ru-nico** in rigid matrices, where ligand dissociation from the metal complex is inhibited as compared to its solution state.

## Results and Discussion

**Figure 1:**
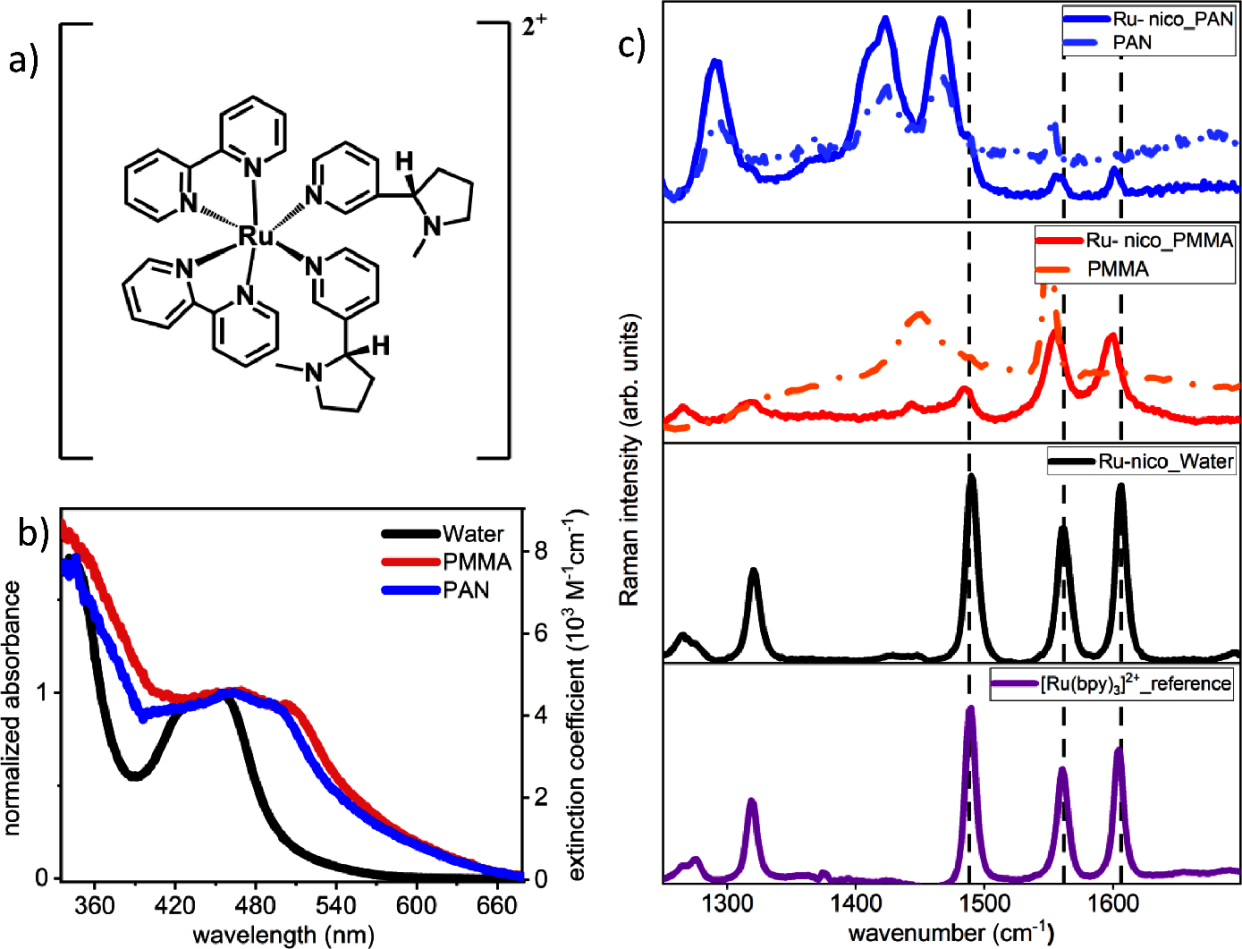
(a) Chemical structure of [Ru(bpy)_2_(nicotine)_2_] (Cl)_2_ (**Ru-nico**). (b) Normalized steady-state absorption spectra of **Ru-nico** complex in water, 1% by mass in film (PMMA) and film (PAN) in the wavelength range of 340–670 nm. The spectra are normalized to the maximum of the visible absorption band. The right axis in steady-state absorption spectra in (b) provides the extinction coefficient values of the **Ru-nico** in aqueous solution. (c) Resonance Raman spectra of the **Ru-nico** recorded upon excitation at 405 nm, i.e., in resonance with the ^1^MLCT transition. The black spectrum represents the rR spectrum of **Ru-nico** in water, and the purple spectrum corresponds to the reference, i.e., Ru(bpy)_3_]^2+^ dissolved in ACN. The blue and red lines represent the rR spectra of **Ru-nico** in films of PAN and PMMA, respectively, while the dash-dotted blue and red lines reflect the spectrum recorded of PAN and PMMA films, respectively, without the addition of **Ru-nico**.

Figure 1 shows the chemical structure of **Ru-nico** together with its normalized steady-state absorption spectrum in aqueous solutions and polymer films. At 455 nm, the ^1^MLCT band typical for Ru(II)-polypyridine complexes is visible together with a band at 345 nm, which is attributed to a ^1^MLCT transition involving acceptor orbitals localized on the nicotine ligand.^38,39^ When embedding **Ru-nico** in PMMA and PAN films, the MLCT absorption band broadens and its maximum shifts to about 506 nm. These observations point to intermolecular interactions of the immobilized molecules in the solid films and are reminiscent of the behavior observed for π conjugated systems in films.^40,41^ Chang et al. observed that ([Ru(bpy)_3_]^2+^ and [Ru(bpy)_3_(mcbpy)]^2+^) complexes aggregate when immobilized within polymer matrices due to intermolecular interactions.^50^ This is particularly evident, as they reported increased aggregation with an increased molecular loading. We also see a similar trend as our steady-state electronic absorption measurements reveal a comparable spectral profile between **Ru-nico** in aqueous solution and **Ru-nico** PMMA and PAN films when prepared from a diluted solution. The spectral profile changes significantly when the molecular loading is increased. (see Supporting Information Figure S1) However, the broadening of the peaks indicates an increased inhomogeneity of the environments of the complexes in the polymers compared to solution samples.^42,43^

To further probe the chromophoric properties of **Ru-nico**, resonance Raman (rR) measurements are performed upon irradiation at 405 nm, i.e., in resonance with the MLCT transition. Figure 1c compares the spectra obtained from solution and film samples to the spectrum of the reference compound [Ru(bpy)_3_]^2+^_._ The rR bands of the reference [Ru(bpy)_3_]^2+^ at 1318, 1485, 1560, and 1604 cm^–1^ also dominate the spectra of **Ru-nico**, irrespective of the complex’s environment. Thus, the experiments evidence that the **Ru-nico** absorption band at 405 nm is due to an Ru (II) → bpy-^1^MLCT transition and that changes in the local environment of the complexes and possible aggregation in the polymer films do not perturb the complexes’ geometry to an extent that the nature of the Franck–Condon active vibrations coupled to the ^1^MLCT transition at 405 nm is altered.^44,45^

### Emission Spectroscopy

**Figure 2:**
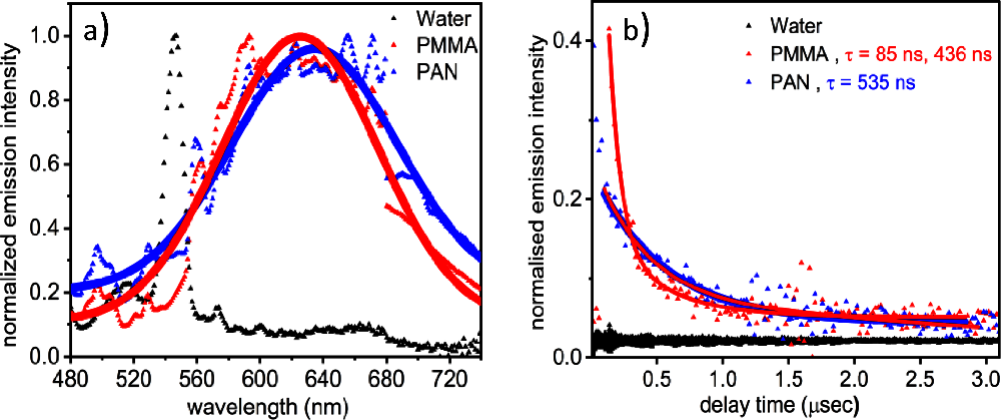
(a) normalized emission spectrum at the excitation wavelength of 450 nm of **Ru-nico** complex in water, film (PMMA), and film (PAN) in the wavelength range of 480–740 nm. The dots denote the emission observed in the films, but because of scattering, it was fitted with a Gaussian line shape denoted by lines. (b) normalized integrated emission decay kinetics at the excitation wavelength of 450 nm of **Ru-nico** complex in water, film (PMMA), and film (PAN) in the whole wavelength range of 550–740 nm with respect to their delay time in μs.

Figure 2 depicts (normalized) emission spectra of the **Ru-nico** complex in different environments upon excitation at 450 nm. While the complex is virtually nonemissive in water, ^3^MLCT emission is observed from **Ru-nico** embedded into the polymers. Due to the inhomogeneities of the polymer samples, scattering is superimposed on the emission signal, so in order to guide the eye, the emission spectra displayed in Figure 2 were fitted to a Gaussian function. This fit is displayed together with the experimental data. When embedded into PMMA and PAN, the emission of **Ru-nico** is found to peak at 625 and 635 nm, respectively. The rather small environmentally induced shift of the emission maximum, i.e., 252 cm^–1^, is attributed to the different dielectric constants of PMMA and PAN.^46−48^ While the emission spectrum of **Ru-nico** in polymer films resembles the features of the benchmark complex [Ru(bpy)_3_]^2+^.^49^ The emission quenching in water relates to the light-induced ligand-loss chemistry of **Ru-nico**. This process appears hindered when the complex is embedded into the polymer matrices rendering the complex emissive.

The ^3^MLCT emission decay of the **Ru-nico** is depicted in Figure 2b. Both in PMMA and PAN, a slow decay component with a characteristic time constant in the order of 0.5 μs is observed. Thus, when embedding the photolabile **Ru-nico** in polymer matrices, its emission lifetime becomes comparable to that of the benchmark [Ru(bpy)_3_]^2+^ in PMMA, which was determined to be 1.8 μs.^33^ This indicates that the restricted intramolecular flexibility in the absence of (liquid) solvent destabilizes the metal-centered states and consequently increases the energy gap between the ^3^MLCT and the ^3^MC states. This increased energy gap facilitates radiative decay and can be assigned to the phosphorescence from the ^3^MLCT state.^33,51−54^ In addition, PMMA gives a second decay component with τ = 85 ns required to fit the data. Chang et al. observed similar multiexponential decay components in [Ru(bpy)_3_]^2+^ in PMMA films as τ = 5, 70, and 220 ns. They describe it as self-quenching kinetics because of aggregation in the PMMA film compared to a monoexponential decay (τ = 700 ns) in ethanol solution.^50^

### Femtosecond Transient Absorption Spectroscopy

**Figure 3:**
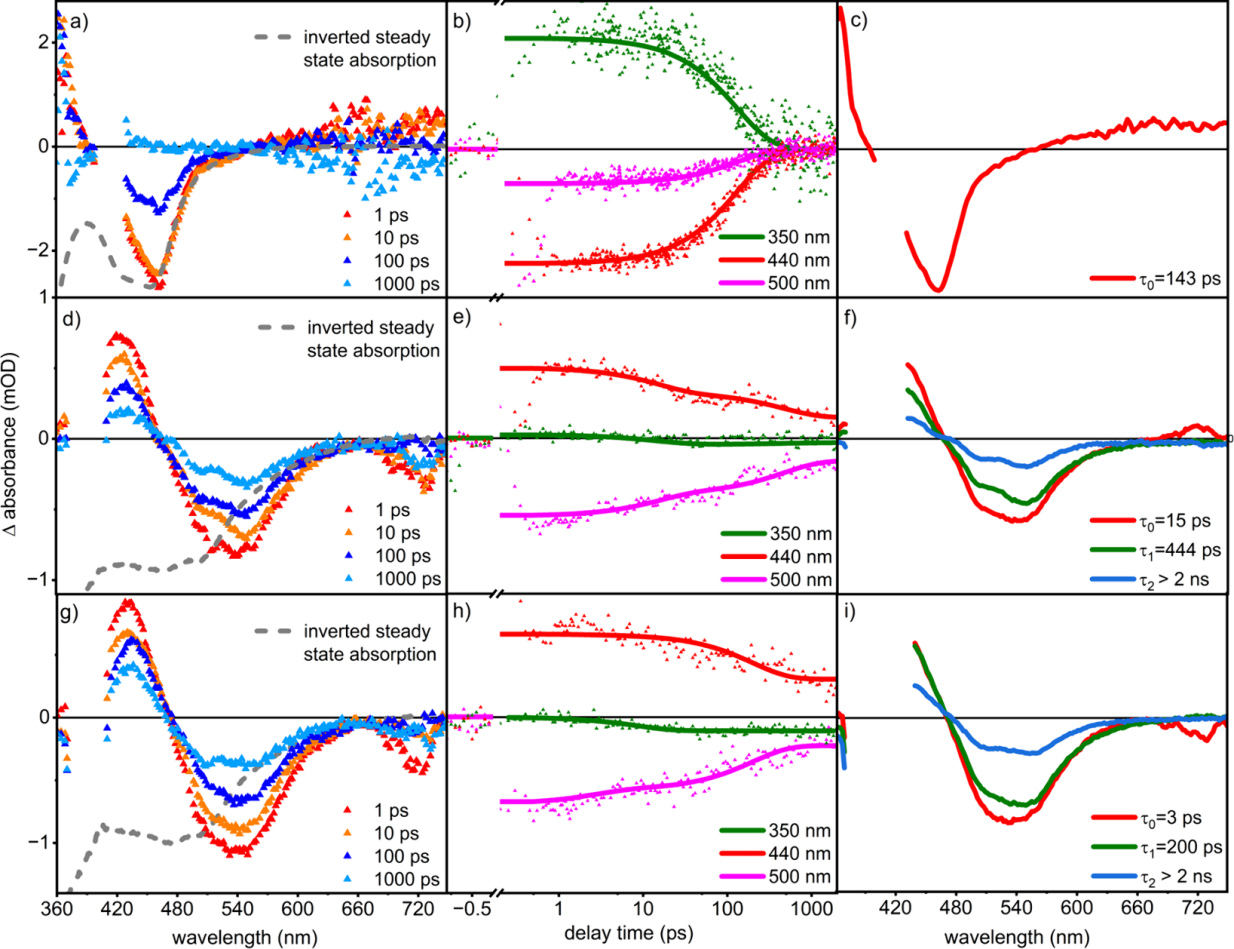
(a), (d), and (g) show the transient absorption spectrum of **Ru-nico** complex in water, film (PMMA), and film (PAN), respectively, in the wavelength range of 340–750 nm. (b), (e), and (h) show the kinetics with respect to their delay time in ps. (c), (f), and (i) depict the decay-associated spectra (DAS) in the wavelength range of 350–740 nm.

Figure 3 displays the transient absorption data of the **Ru-nico** recorded upon excitation of the ^1^MLCT transition at 400 nm. Irrespective of the environment of **Ru-nico,** an initial rise of the transient absorption signal is followed by a decay on ps to ns time scales. Notably, the signal decays without any discernible spectral changes. However, the actual shape of the differential absorption signal varies with the environment. When studying **Ru-nico** in an aqueous solution, the ground state bleach peaks at ca. 480 nm and is accompanied by a weak, unstructured excited state absorption (ESA) in the red part of the spectrum and a prominent ESA band at 360 nm. The latter stems from ππ* transitions within the reduced bpy ligand, while the broad ESA band at long probe wavelengths is due to LMCT transitions from the reduced bpy ligand to the Ru(III) ion.^39,55−57^ These transient absorption features decay to zero within the 2 ns delay time range accessible in the experiment, and no appreciable photoproduct formation is observed, e.g., [Ru(bpy)_2_(nicotine)(H_2_O)]^2+^. Instead, kinetic analysis based on the global exponential fitting of the data reveals the presence of a single kinetic process with a characteristic rate constant of 143 ps^–1^. This process manifests itself as a synchroneous decay of the entire transient absorption signal. This observation taken together with the nonemissive nature of **Ru-nico** in water, which we ascribe to light-driven ligand loss, we conclude that the ligand dissociates only fleetingly, and geminate recombination occurs rapidly.^58−60^

The absence of any photoproduct absorption is somewhat unexpected, as Greenough et al. as well as Schüssler et al. have observed pentacoordinated Ru-nicotinamide intermediates in solution as a result of light-initiated nicotinamide ligand loss. Coordination of a water molecule to the penta-coordinate intermediate yielded the hexa-coordinated Ru-nicotinamide-aqua complex with a characteristic absorption band at 480 nm.^38,61,62^ Such absorption is not observed for **Ru-nico**. Thus, we conclude that the labilization of the Ru-nicotine bond upon population of the ^3^MC state is followed by rapid geminate recombination, repopulating the electronic ground state of the parent complex.

In Ru(II) polypyridine complexes with monodentate ligands such as nicotine, nicotinamide, and pyridine, the electron-withdrawing ability of the ligand is modulating the ligand dissociation reaction by influencing the strength of the metal–ligand bond. For instance, the electron-withdrawing amide group in the nicotinamide ligand weakens the Ru–N(nicotinamide) bond as compared to, e.g., the nicotine ligand. As a result, dissociation of a nicotine ligand might not occur readily on an ultrafast time scale. Furthermore, the acidic proton in nicotinamide can participate in intramolecular hydrogen bonding with water molecules, particularly upon keto–enol tautomerization. This hydrogen bonding might provide a pathway for water molecules to enter the complex and facilitate the formation of the aqua-complex intermediate. As the nicotine ligand lacks acidic hydrogen, we hypothesize that **Ru-nico** is less prone to the formation of the spectroscopically identifiable intermediate compared to its nicotinamide counterpart.^63,64^

Comparison of the transient absorption data of **Ru-nico** in aqueous solution to those observed in PMMA or PAN films reveals distinct spectral-temporal differences. For **Ru-nico** in PMMA films, the maximum GSB is observed at 550 nm, corresponding to a red shift of 2134 cm^–1^ with respect to the GSB for the complex in solution. Additionally, a sharp ESA at 440 nm is observed in TA data recorded for **Ru-nico** in the PMMA film. This ESA band originates from π–π* transitions from reduced bpy ligand but with a red shift of the ESA band from an aqueous solution at 360 nm. This red shifting of ESA possibly could be because of induced aggregation in polymer films or difference in the dielectric constants of the polymers.^41,42,65^ Notably, the overall differential absorption signal does not decay within the experimentally accessible delay-time window of 2 ns. The long-lived differential absorption signal aligns with the emission observed for **Ru-nico** when embedded into the polymer. The excited-state relaxation processes leading up to the long-lived (emissive) excited state are accounted by a biexponential relaxation model. A global fit of the transient absorption data yields the characteristic rate constants with one short-lived (fast rate) as *k*_0_ = 3 ps^–1^ which is assigned to the vibrational cooling in the system followed by another longer lived component of ^3^MLCT excited state with *k*_1_ = 444 ps^–1^. The rate for vibrational cooling observed here is reminiscent of work by Chang et al., who studied the light-induced processes in [Ru(bpy)_3_]^2+^ both in ethanol solution and in PMMA films.^50^ The spectral changes associated with the rate *k*_1_ = 444 ps^–1^ resemble a loss of the MLCT-ground state bleach and the characteristic π–π* absorption band at 440 nm. Hence, we conclude that this process is associated with a (fractional) loss of ^3^MLCT population, likely associated with a fraction of the complexes undergoing ligand loss followed by rapid recombination, as the rather rigid polymer environment the molecular fragments, i.e., the isolated ligand and the pentacoordinated complex fragment, cannot be individually stabilized by solvation.

Nonetheless, the experimental data presented here suggest that, for a large fraction of **Ru-nico** complexes in PMMA, the ^3^MLCT state is long-lived and decays radiatively to the ground state. The rigidity of the polymer matrix apparently imposes constraints on the intramolecular reorganization of the photoexcited complex, preventing the population of the dissociative ^3^MC state. Similar experimental findings are obtained for **Ru-nico** in a PAN matrix (see Figure 3g). However, by changing the polymer, slight changes in the rates of the light-driven processes are notable: *k*_o_ changes from 3 ps^–1^ for PAN to 6 ps^–1^ for PMMA, while the fractional subns decay of the ^3^MLCT state, the rate of 444 ps^–1^ in PMMA is slightly higher than in PAN. These differences in rates are associated with slight changes in the dielectric constant of the polymer films and hence slightly different energetic stabilization of the ^3^MLCT relative to the ^3^MC states. The simultaneous presence of a long-lived emissive ^3^MLCT state and a short-lived MLCT component (associated with k1) indicates that the polymer matrices provide an inhomogeneous environment to **Ru-nico**. This in turn causes about 60% of the excited complexes to decay via ligand loss and subsequent rapid geminate recombination as judged by the magnitude of ground state recovery on a subns time scale. At the same time, about 40% of the complexes form a long-lived emissive state bypassing this photochemical deactivation channel.

## Conclusions

This contribution compares the light-induced processes of a [Ru(bpy)_2_(nicotine)_2_] (Cl)_2_ (**Ru-nico)** upon MLCT excitation at 405 nm in solution as well as in polymer films, i.e., PMMA and polyacrylonitrile (PAN). In solution, the complex is nonemissive, and ground-state recovery occurs with a rate constant of 143 ps^–1^. However, when embedded into the polymer matrices, the complex’s emission switches on, which goes hand in hand with the formation of a long-lived ^3^MLCT state as determined by transient absorption spectroscopy. We ascribe this behavior to hindered ligand loss in the rigid polymer matrix, which impacts intermolecular reorganization in the complex and hence prevents the population of the dissociative ^3^MC state. Instead, an emissive ^3^MLCT state dominates the excited-state relaxation of **Ru-nico** in the polymer films. Different photophysics (chemistry) of the molecules in solution and in rigid medium opens up a new avenue to study the charge transfer dynamics and ligand functionalization in different local environments.

## Experimental Section

[Ru(bpy)_2_(nicotine)_2_] (Cl)_2_ (**Ru-nico**) complex was purchased from Sigma-Aldrich and used without any further purification. Two 500 μM stock solutions of the **Ru-nico** were prepared in chloroform and (DMF) because of the solubility in different polymers. These solutions were used to prepare 1% **Ru-nico** by weight in PMMA and PAN polymers using chloroform and DMF solvents, respectively. For preparing the **Ru-nico** thin film in a PMMA matrix, silica-coated glass substrates were used. **Ru-nico** film with PAN matrix was prepared directly on the glass substrate. The 1 mm thick glass substrates were ultrasonically cleaned with water and acetone for 5–10 min and then dried in an oven before the deposition of the polymer films.

In polymers, counterions such as Cl^–^ may remain closely associated with the Ru(II) complex since the films are prepared by drop-casting the complex from the solution where the anions surround the Ru center in solvated forms. From our photoirradiation experiment (please see the Supporting Information, Figure S2), we observed that the photodissociation of nicotine in solution leads to the aqua product of ([Ru(bpy)_2_(nicotine)_2_]Cl_2_) as ([Ru(bpy)_2_(nicotine)(H_2_O)]Cl_2_) within 5 min of irradiation. However, we do not observe any photodissociation of Ru-nico in polymers even after 12 h. In any case, we do not observe counteranion-associated product that has been seen by Schilter et al. via collision-induced dissociation.^66^

### Steady State Spectroscopy

Steady-state absorption spectra were obtained by using a Jasco V780 UV/vis/NIR spectrophotometer with a 10 mm quartz cuvette. Absorption spectra of polymer films were recorded from films coated on glass substrates of 10 mm × 10 mm size.

### Time-Resolved Spectroscopy

Transient absorption experiments were performed using a previously reported setup.^67,68^ The solution samples were prepared to yield an optical density of 0.2 at the excitation wavelength, i.e., 400 nm, in a 1 mm path length quartz cuvette. The pump power was adjusted to 0.4 μJ/pulse. To obtain transient absorption data from the film samples, the pump power was increased to 1.5 μJ/pulse. To check for sample degradation during the spectroscopic experiments, the UV–vis spectra of both the solution and film samples were monitored before and after each experiment. No changes in the absorption properties were discernible.

The transient absorption spectrometer employed in this study is based on an amplified Ti:Sapphire laser, the output of which is split to generate pump- and probe–pulse trains. The pump-pulses are generated by second harmonic generation in a BBO crystal while the supercontinuum probe is generated by focusing a fraction of the amplifier output into a rotating CaF_2_ plate. The mutual polarization of pump- and probe–pulses is set to a magic angle and the pulses are overlapped at the sample position. The transmitted intensity of the probe pulse is detected on a diode array detector as a function of the temporal delay between the pump- and the probe–pulses.

The transient absorption signals are processed by using the KiMoPAck Python tool (chirp and background correction). The fitting of the chirp-corrected file was obtained by performing a global fit in the data. To omit coherent artifacts in the data analysis, the data recorded in the delay time of 100 fs was removed during multiexponential fitting of the data. The decay-associated spectra (DAS) obtained after the data fitting represent the fit of the decaying of the spectral features with a given time constant and are obtained by global fitting analysis of all the wavelengths in the transient data. The DAS provides detailed information on the excited-state dynamics of the complex as the formation, decay, and interconversion of states. Changes in decay time and spectral features in DAS can identify the effects of solvents and aggregation in a system.^69,70^

Nanosecond transient emission spectroscopy measurements were obtained from the previously described set up.^71^ The pump pulse was obtained at the excitation (OPO used for excitation) of 450 nm by using a ND:YAG laser (Surelite) with a repetition rate of 10 Hz. The transient signals were obtained by using a detection system from Pascher instruments AB, Lund, Sweden, and the optical density for the solution was maintained at 0.4 at the excitation wavelength. For the solution, a 10 mm quartz cuvette was used at the excitation, whereas for films, 10 mm × 10 mm coated films on a glass substrate were used. The integrity of the sample was ensured by measuring UV–vis before and after the measurements for each of the samples. The instrument response function is measured to be 50 ns in our measurement condition (please see the Supporting Information in Figure S3).

In order to allow an appropriate computational description of the ([Ru(bpy)_2_(nicotine)_2_]Cl_2_) (**Ru-nico**) complex’s electronic structure as well as to account for explicit interactions with the PMMA polymer backbone, multiscale approaches, such as QM/MM and ONIOM, are the methods of choice. Ideally, these simulations are coupled with molecular dynamics to map the potential energy landscape of the hybrid system. Such simulations allow to address the complicated electronic structure of the photoactive subunit by a high-level quantum chemical method and account for its interaction with a (rigid) polymer subsystem.^72^ Unfortunately, such an extensive computational investigation is well beyond the scope of the present contribution.

### Resonance Raman Spectroscopy

Resonance Raman spectra were measured upon excitation at 405 nm. The excitation light was provided by a diode laser, which delivered a power of 8 mW to the sample. The Raman signal was collected after passing a long pass filter of 405 nm (Semrock, USA) in an IsoPlane 160 grating spectrometer (Princeton Instrument, USA) with a slit width of 50 μm and a 1200 grooves/mm grating.

## Supporting Information Available

Supporting Information contains chemical structure of the [Ru(bpy)_2_(nicotine)_2_] (Cl)_2_ (**Ru-nico**) complex, photoirradiation experiment of the **Ru-nico** complex in water and normalized emission spectra with blank glass substrate (PDF)
