# Supplementary material for: An Increase in the Rigidity of the Environment Favors MLCT over the MC State in [Ru(bpy)2(Nicotine)2](Cl)2: A Case Study of Photolabile Ligands
Source: J Phys Chem A. 2024 Nov 4;129(2):439–46. doi: 10.1021/acs.jpca.4c04914 (PMC11744796; doi:10.1021/acs.jpca.4c04914)
Supplement: Supplementary file 1 — jp4c04914_si_001.pdf [file jp4c04914_si_001.pdf]

Supplementary Information

An increase in the rigidity of the environment favors MLCT over MC state in $[Ru(bpy)_2(nicotine)_2](Cl)_2$ - A case study of photolabile ligands

Mohini Semwal, [a,b] *Nikita Vashistha,* [a,b] *Sven Rau,* [c] *Benjamin Dietzek-Ivanšić* [*a,b]

[*]Corresponding author

Email: benjamin.dietzek@uni-jena.de

[a] Friedrich Schiller University Jena, Institute of Physical Chemistry, Helmholtzweg 4, Jena, Germany

[b] Leibniz Institute of Photonic Technology, Research Department Functional Interfaces, Albert-Einstein-Str. 9, Jena, Germany

[c] Institute for Inorganic Chemistry I, Albert-Einstein-Allee 11, 89081 Ulm, Germany

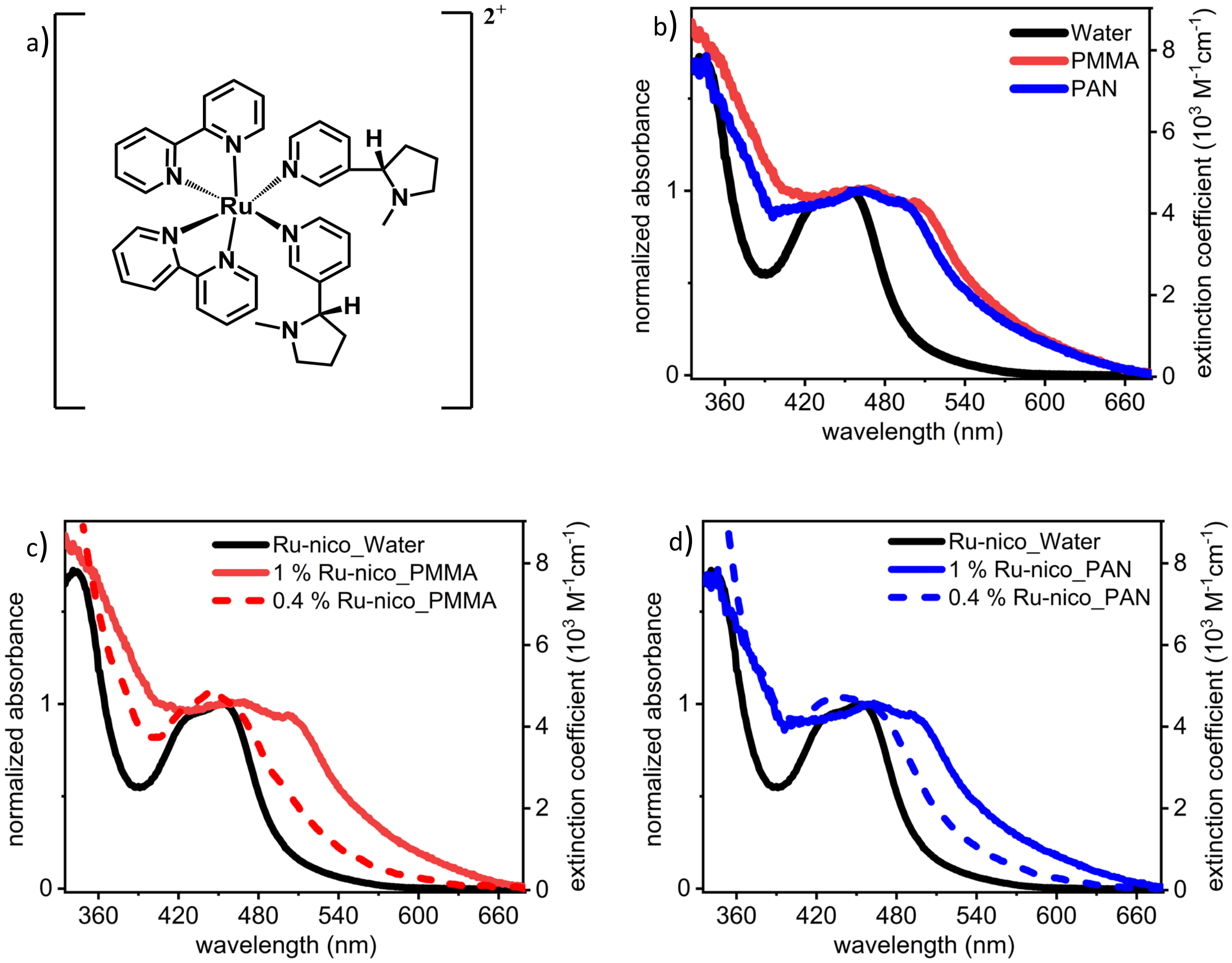

Figure S1 a) Chemical structure of [Ru(bpy)$_2$(nicotine)$_2$] (Cl)$_2$ **(Ru-nico)**. b) normalized steady state absorption spectra of **Ru-nico** complex in water, 1% by mass in film (PMMA) and film (PAN) in the wavelength range of 340 to 670 nm. The spectra are normalized to the maximum of the visible absorption band. The right axis in steady state absorption spectra in b) provide the extinction coefficient values of the **Ru-nico** in aqueous solution. c) normalized steady state absorption spectra of **Ru-nico** complex in water, 0.4% by mass in film (PMMA) compared with 1% by mass in film (PMMA) in the wavelength range of 340 to 670 nm. normalized steady state absorption spectra of **Ru-nico** complex in water, 0.4% by mass in film (PAN) compared with 1% by mass in film (PAN) in the wavelength range of 340 to 670 nm.

The electronic absorption measurements reveal a comparable spectral profile between **Ru-nico** in aqueous solution and **Ru-nico** PMMA and PAN films when prepared from a diluted solution. The spectral profile changes significantly when the molecular loading is increased. In the absorption band ^{1}MLCT band typical for Ru(II)-polypyridine complexes is visible at 455 nm in aqueous solution for **Ru-nico** while the absorption band broadens to 506 nm when embedded in the PMMA and PAN films of 1% **Ru-nico**.

However, if the molecular loading of **Ru-nico** is decreased to 0.4 % the absorption profile of PMMA and PAN films becomes significantly comparable to the absorption profile of **Ru-nico** in aqueous solution. The ^{1}MLCT band now observed at 458 nm with a peak broadening at 458 nm comparable to 455 nm MLCT band of **Ru-nico** in aqueous solution. The peak broadening in films is however due to the inhomogeneity of the polymer films prepared by drop casting method.

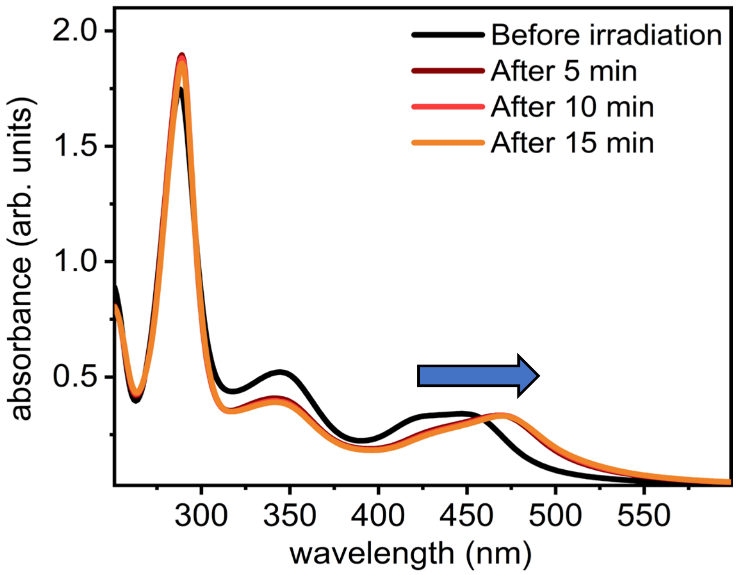

Figure S2 Photoirradiation of **Ru-nico** in water at room temperature irradiated with one LED-stick (405 nm with power of 20mW) in the wavelength range of 250 to 600 nm.

In a continuous wave (CW) photoirradiation experiment of **Ru-nico** in aqueous solution with very high power (30 mW), we observed an increase in absorption band at 480 nm and decrease of the MLCT band at 450 nm within 5 minutes of CW irradiation. This is attributed to the formation of aqua product of $[Ru(bpy)_2(nicotine)(H_2O)]^{2+}$. However, we do not observe any photodissociation of **Ru-nico** in polymers even after 12 hours.

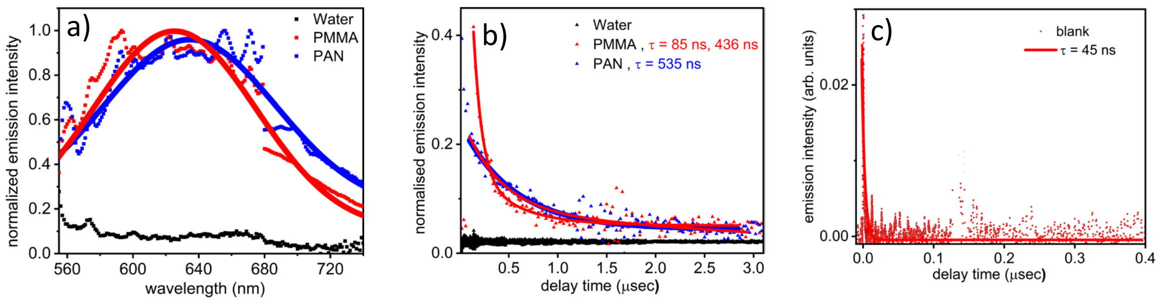

Figure S3 a) normalized emission spectrum at the excitation wavelength of 450 nm of **Ru-nico** complex in water, film poly(methyl-methacrylate) (PMMA) and film polyacrylonitrile (PAN) in the wavelength range of 550 to 740 nm. The dots denote the emission observed in the films but because of scattering it was fitted with gaussian line shape denoted by lines. b) normalized integrated emission decay kinetics at the excitation wavelength of 450 nm of **Ru-nico** complex in water, film (PMMA) and film (PAN) in the whole wavelength range of 550 to 740 nm with respect to their delay time in μs. c) blank glass substrate decay kinetics measured before the sample.
